# Influence of Slaughter Weight and Sex on Growth Performance, Carcass Characteristics and Ham Traits of Heavy Pigs Fed Ad-Libitum

**DOI:** 10.3390/ani12020215

**Published:** 2022-01-17

**Authors:** Isaac Hyeladi Malgwi, Diana Giannuzzi, Luigi Gallo, Veronika Halas, Paolo Carnier, Stefano Schiavon

**Affiliations:** 1 Department of Agronomy, Food, Natural Resources, Animals and Environment (DAFNAE), University of Padova, Viale dell’ Università 16, Legnaro, I-35020 Padova, Italy; luigi.gallo@unipd.it (L.G.); stefano.schiavon@unipd.it (S.S.); 2 Department of Farm Animal Nutrition, Hungarian University of Agriculture and Life Sciences (MATE), Kaposvár Campus, Guba Sándor Utca 40, H-7400 Kaposvár, Hungary; halas.veronika@uni-mate.hu; 3 Department of Comparative Biomedicine and Food Science (BCA), University of Padova, Agripolis, Viale dell’Università 16, Legnaro, I-35020 Padova, Italy; paolo.carnier@unipd.it

**Keywords:** pigs, slaughter weight, ham quality, feed efficiency, carcass quality, sex

## Abstract

**Simple Summary:**

In recent years, pigs involved in the dry-cured ham production system have suffered from excessive leanness. This has led to the increase of slaughter weight (SW) to achieve greater carcass and ham fatness statuses to compensate for the loss in dry-curing aptitude. The production guidelines for the production of Italian dry-cured ham are currently under revision and an extension of the range of carcass weights from 126 to 168 kg, corresponding to about 146 to 210 kg of SW, has been proposed. However, little is known about the influence of SW in the range of 140–200 kg on growth performance, feed efficiency, carcass quality and ham curing aptitude. We hypothesized that an increased SW could exert a positive influence on ham characteristics. Data from 159 pigs fed ad libitum with diets, unlimiting for nutrient contents, up to 8 or 9 months of age (140–200 kg SW) were used. Greater SWs were linearly and positively associated with the growth performance of the pigs and with better ham quality traits. Greater SW increased ham weight, muscularity, and greater fat covering, according to the dry-cured ham industry’s expectations. Barrows produced hams with greater weight and marbling than gilts.

**Abstract:**

Slaughter weight (SW) is critical for dry-cured ham production systems with heavy pigs. A total of 159 C21 Goland pigs (gilts and barrows) at 95 ± 9.0 kg body weight (BW) from three batches were used to investigate the impact of ad libitum feeding on SW, growth performance, feed efficiency, and carcass and green ham characteristics. Diets contained 10 MJ/kg of net energy and 7.4 and 6.0 g/kg of SID-lysine. Slaughter weight classes (SWC) included <165, 165–180, 180–110 and >210 kg BW. In each batch, pigs were sacrificed at 230 or 258 d of age. Left hams were scored for round shape, fat cover thickness, marbling, lean colour, bicolour and veining. Data were analyzed with a model considering SWC, sex and SWC × Sex interactions as fixed factors and the batch as a random factor. The linear, quadratic and cubic effects of SWC were tested, but only linear effects were found. Results showed that pigs with greater SWC had greater average daily gain and feed consumption, with similar feed efficiency and better ham quality traits: greater ham weight, muscularity, and fat coveringin correspondence of *semimembranosus* muscle. Barrows were heavier and produced hams with slightly better characteristics than gilts.

## 1. Introduction

The major limitation for increases in pig slaughter weights (SWs) is increases in carcass adiposity and the worsening of feed efficiency with increasing SW [1]. However, in the last few decades, genetic improvements have determined a strong increase in feed efficiency and a production of very lean carcasses with a limited amount of fat. As a consequence, a progressive increase in pig SW has been observed in many countries [1]. Cisneros et al. [2] have indicated that modern, high-lean-gain genotypes have the potential to be slaughtered at heavier weights with less effect on carcass merit and (or) feed conversion efficiency compared with low-lean-gain genotypes. Indeed, they concluded that modern genotypes can be slaughtered at live weights up to 160 kg with limited impact on growth performance, commercial meat yields or meat quality characteristics [2].

For dry-cured ham production, adequate fat covering and marbling are required, so the pigs must be slaughtered at heavy weights, often greater than 130 kg [3,4]. In these production conditions, an increase in SW is considered a potential strategy to compensate for the increased leanness of modern pig genotypes [5]. In the Italian dry-cured ham production circuits, an SW of 160 ± 16 kg and a minimum age of nine months are indicated by the official production guidelines [6]. To comply with these prescriptions with modern pig genotypes, restricted feeding is required [7]. However, this is an inefficient strategy, and a progressive increase in SW has also been observed in this production system INEQ [8]. Therefore, a revision of these guidelines is required and an extension of the range of carcass weights from 120 to 168 kg, corresponding to about 146 to 210 kg of SW, has been recently proposed to the authorities.

The extension of the admitted SW range implies the possibility of adopting an ad libitum feeding strategy that would better exploit the genetic potential of individual pigs for growth—although with a reduction of body and carcass uniformity among pigs of the same batch [9,10]. There are not many studies that have considered the influence of increased SW on growth performance, feed efficiency, and carcass and ham characteristics in such body weight (BW) intervals. In addition, previous studies that have considered the effect of SW [3,11,12] have shown confounded effects between SW and age at slaughter, and only sporadically have the two effects been separately evaluated, highlighting their diverse implications [13].

Assuming that pigs selected for dry-cured ham production are slaughtered at about the same age, it can be hypothesized that those heavier at slaughter would be those with greater feed consumption, growth rate and carcass and ham weight, but also with greater carcass adiposity, ham marbling and ham fat covering. In addition, some loss in feed efficiency may occur, increasing the SW [1]. However, such responses would depend on the propension of the pig genotype for lean and fat deposition at heavy weights.

This paper aimed to study, in groups of pigs fed ad libitum and selected for dry-cured ham production, the relationships between SW, growth performance, feed efficiency, and carcass and green ham characteristics.

## 2. Materials and Methods

### 2.1. Pig Housing, Rearing and Slaughtering

The data used in this research originated from a previous experiment that involved 336 pigs, from three batches of 112 pigs each [5]. Briefly, the pigs of Malgwi et al. [5] were divided into four experimental groups. The study, arranged as a split-plot design with sex within a pen, included four (4) feeding groups representing four (4) alternative rearing strategies. Only the two groups of pigs (for a total of 168 individuals) fed ad libitum high protein diets, not limiting for the indispensable amino acid content, were used for the purposes of current research. Such non-limiting conditions were applied to exploit the pigs’ potential for protein and lipid deposition [14,15]. Among these two groups, the first represented a rearing strategy aimed at reaching 170 kg SW at the minimum possible age, which was in the order of 8 months (younger age, YA). The second represented a strategy aimed at reaching the maximum SW (>170 kg) at nine months’ slaughter age (greater weight, GW). Pigs of this group were fed ad libitum the same high protein feeds of the YA group, and at slaughter the pigs were about 190 kg SW. During the test, nine animals were moved to the infirmary and excluded for health problems, for a final number of 159 individuals.

Pigs were members of 68 full-sib families of the C21 Goland boar line (Gorzagri, Fonzaso, Italy), generated by mating 13 boars to 67 sows. Besides growth and residual feed efficiency, the breeding goal of the C21 Goland line includes traits related to the quality of raw ham [16] and its suitability for dry-curing [17]. All pigs were born in the same week, they were reared on the same farm and fed the same commercial diets until their transfer to the experimental station at 95 ± 9.0 kg BW. The pigs were housed in pens in groups of 14 pigs, with barrows and gilts mixed in equal proportion in the same pen. An across-batch rotation scheme was used to assign each treatment group to a given pen in different batches. Each pen (5.8 × 3.8 m, fully slatted floors) was equipped with a single-space electronic feeder (Compident Pig–MLP, Schauer Agrotronic, Prambachkirchen, Austria). The feeding station recorded, daily and on an individual basis, feed intake and other behavior traits [18]. 

### 2.2. Diets and Feeding

In early (90 to 120 kg BW) and late (120 kg BW upwards) finishing periods, the pigs received cereal–soybean meal-based diets (Table 1). The feeds were formulated to contain 10 MJ/kg of net energy without limiting the indispensable amino acid content, with 7.4 and 6.0 g/kg SID-lysine considered the first limiting amino acid [19]. Feeds were manufactured by the Progeo Feed Industry. Water was accessed freely from nipple drinkers within each pen. The major details of the nutritional characteristics of the feeds are given in [5]. 

At the start of the experiment, and the day before slaughtering, the pigs were weighed with a scale. The pigs of the YA and GW groups were reared in the same way, but they were slaughtered at different ages. These two groups had homoscedastic variances and ample variations in SW.

### 2.3. Slaughter and Evaluation of Carcass and Green Ham Traits

The pigs of the YA and GW groups were slaughtered, on average, after 85 or 116 days on feed—corresponding to almost 8 or 9 months of age. An extra month of feeding would increase the SW, the daily and cumulated feed consumption, the carcass and the ham fat covering, the ham size, and would reduce the average daily gain and feed efficiency. Slaughter and carcass dressing were carried out as described in [20]. Hot carcass weight was recorded online, and the lean percentage was estimated by image analysis of the left carcass side (CSB-Image-Meter, CSB-System AG, Geilenkirchen, Germany), as guided by [21,22]. Carcass weight was measured as the head-on weight, as is currently practised in Italy and Canada [23]. Loin with ribs, shoulder, thigh, lard and belly were weighed about 1 to 3 h after slaughter using an electronic scale. Green hams were chilled (0–2 °C) for 24 h, trimmed and weighed again. A trained operator scored all left hams as described in [24] for round shape (0 = low to 4 = high, optimum: 1 to 2), fat cover thickness ( −4 = very thin to 4 = very thick, optimum: 0 to 1), marbling (0 = absent to 4 = very evident, optimum: 1), lean color ( −4 = very pale to 4 = very dark, optimum = 0), bicolor (0 = absent to 4 = very evident, optimum = 0) and veining (0 = absent to 4 = very evident, optimum = 0). A reference standard was used at the beginning of each of nine scoring sessions. The scoring sessions were performed by placing the hams on a table with a plastic white surface, all placed in the same room illuminated with artificial lamps. To limit the influence of personal subjective factors, a single operator with decades of experience in scoring ham for the genetic improvement of the Goland C21 pig line was involved. Comparable scoring grids for these traits have also been reported by others [25,26,27]. The subcutaneous fat depth of the green ham was measured in the proximity of the muscles *biceps femoris* (P1) and *semimembranosus* (P2) using a ruler or a portable ultrasound system (Aloka SSD 500 equipped with UST-5512 7.5 MHz linear transducer probe, Hitachi Medical Systems S.p.A., Milan, Italy), respectively.

Hams were moved to the ham factory within two days after the slaughter, where they were trimmed again and weighted. The hams were trimmed to obtain the typical shape of Veneto ham, without the trotter.

### 2.4. Statistical Analysis

According to current guidelines, 160 kg ± 10% is the average weight of the batch. Accordingly, data were grouped into four SW classes (SWC), with about 20 kg SW of difference between one class and the following one. The first SWC represented pigs with lighter SW (<165 kg SW), which is still in agreement with current guidelines. The second SWC (165−180 kg SW) were somewhat heavier pigs, with SWs similar to those frequently found in practice. The third SWC represented pigs with SWs (>180, <210 kg SW) in agreement with the proposal of the guideline revisions, and the fourth SWC (>210 kg) represented pigs that were too heavy and would be discarded if the new production guidelines proposal are approved. 

Carcass and ham trait data were analyzed using a GLM procedure in SAS (SAS Inst. Inc., Cary, NC, USA) using the following linear model:

y*_ijkl_* = µ + SWC*_i_* + Sex*_j_* + (SWC × Sex)*_ij_* + Batch*_k_* + e*_ijkl_* (1)

where y*_ijklm_* was the observed trait, µ was the overall intercept of the model, SWC was the fixed effect of the *i^th^* class of SW (*i* = 1, …, 4), Sex was the fixed effect of the *j^th^* sex (*j*: 1 = gilts, 2 = barrows), (SWC × sex) was the interaction effect between the SWC class and sex, Batch was the random effect of the *k^th^* batch (*k* = 1,…,3), and e_ijkl_ was the random residual. 

The Batch and the residuals were assumed to be independently and normally distributed, with a mean of zero and a variance of σ^2^_l_ and σ^2^_e_, respectively. SWC, Sex, and SWC × sex interaction effects were tested in relation to the residual variance (individual). Three degrees of freedom of SWC were used to test the linear, quadratic and cubic effects of increasing SWC. As the quadratic and the cubic components were never significant, the *p*-values of these components were omitted from the tables.

Allometric relationships (y = ax^b^) relating carcass weight to SW, and lean and fat masses to carcass weight were fitted using a spreadsheet.

## 3. Results

### 3.1. Growth Performance and Main Carcass Characteristics

As expected, the lighter SWC were represented in greater proportion by YA pigs, and the heavier SWC by the GW pigs. The most frequent class was the third, followed by the second, the first and the fourth (Table 2). Pigs with the lightest BW at the beginning of the experiment were those that attained the lightest SW. Indeed, initial BW, feed intake and average daily gain increased with increasing SWC (*p* < 0.001), but feed efficiency (gain: feed) did not (*p* = 0.53). 

**Figure 1:**
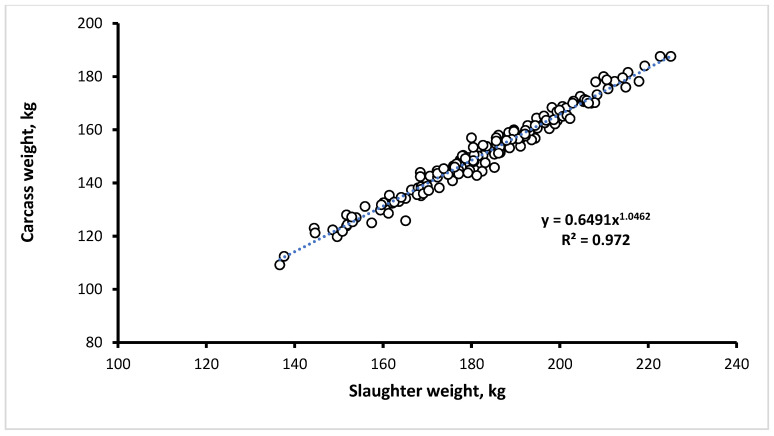
Allometric relationship between the slaughter and carcass weights of ad libitum-fed heavy pigs (*n* = 159).

Consistently, with increasing SWC carcass weight (*p* < 0.001), carcass yield (*p* < 0.001) and carcass backfat depth (*p* < 0.001) linearly increased, whereas the lean meat percentage decreased (*p* < 0.001). The allometric coefficient relating carcass weight to SW was greater than 1.00 (1.046), as the increase in SW was associated with a more than proportional increase in carcass weight (Figure 1).

**Figure 2:**
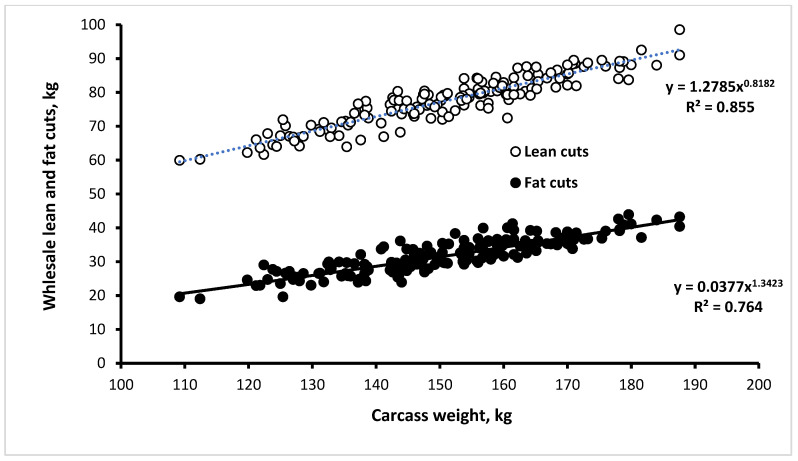
Allometric relationships between lean (shoulders, loins + ribs, and hams) fat (backfat and belly) cuts with carcass weight of ad libitum-fed heavy pigs (*n* = 159).

The weights of lean (*p* < 0.001) and fat cuts (*p* < 0.001) increased with increasing SWC, while the carcass yield of lean cuts decreased (*p* < 0.001), and that of fat cuts increased (*p* < 0.001). The relationships of the lean and the fat cuts on carcass weights evidenced allometric coefficients lower than one (b = 0.855) and greater than one (b = 1.342), respectively (Figure 2).

Sex had little influence on feed intake, average daily gain, SW, carcass weight, carcass yield, carcass backfat depth and total and lean cut weight. The feed efficiency of the barrows was somewhat lower than that of the gilts (*p* = 0.018). However, the barrows had greater SW (*p* = 0.027), carcass yield (*p* = 0.039), fat cuts weight (*p* = 0.043), and lower lean cuts yield (*p* = 0.026). The Sex × SW interaction had negligible influence on growth performance and major carcass traits.

### 3.2. Wholesale Cuts Weights and Proportions 

All the various wholesale cuts weights increased linearly (*p* < 0.001) with increasing SWC (Table 3). However, the yields of all the various lean cuts, i.e., loins and ribs, shoulders, and green and trimmed hams, decreased (*p* < 0001), and those of the fat cuts, back fat and belly increased (*p* < 0.001). The trimming losses increased with increased SWC (*p* < 0.001), both in terms of weight and yield. The barrows had greater belly weight (*p* < 0.001) and yields (*p* = 0.006) than gilts, but lower yields of loins and ribs (*p* = 0.003).

### 3.3. Green and Trimmed Ham Characteristics

The weights of trimmed ham at the slaughterhouse (*p* < 0.001), at the ham factory (*p* < 0.001), and the trimming losses at the ham factory (*p* < 0.001) linearly increased with increased SWC (Table 4). Pigs with greater SWC also had a more round shape (*p* = 0.002), and a greater subcutaneous fat depth in the P2 position (*p* = 0.005). However, the SWC class had little influence on other ham quality parameters.

Sex also had little influence on these ham characteristics, except for marbling and hemorrhage. Barrows had a lower hemorrhage score (*p* = 0.037) and greater marbling (*p* = 0.011) than gilts. The Sex × SWC interaction had no influence on these ham traits.

## 4. Discussion

More than twenty years ago, Cisneros et al. [2] suggested that, for fresh meat production, lean pig genotypes can be slaughtered at live weights up to 160 kg with limited impact on growth performance, commercial meat yields or meat quality characteristics. These authors indicated that increases in SW were associated with increases in feed intake, backfat depth and loin eye area, with minimal changes in growth rate and gain:feed. However, for dry-cured ham production, pigs with greater adiposity compared to those intended for fresh meat consumption are required [1]. This kind of production is conducted according to a variety of systems and is influenced by different climatic environments, rearing and feeding practices, genetic resources, dry curing processes, and market demands and rules indicated by the disciplines of production [27]. In general, hams with insufficient fat covering are inadequate for the dry-curing process, as subcutaneous, intermuscular and intramuscular fat represents a barrier for salt penetration and water diffusion, so that leaner hams are expected to have higher salt contents and lower sensory quality [28]. On the contrary, high levels of fat infiltration were found to be related to softness and pastiness, due to water loss and salt penetration dynamics. Moreover, thick subcutaneous fat covering is undesirable to consumers [27].

The optimal SW and the degree of adiposity of the pigs for dry-cured ham production are strongly affected by the productive context. For instance, [3,29] concluded that an increase in SW up to 124 or 130 kg impairs growth performance and improves some aspects of carcass quality, with few benefits for the Teruel dry-cured ham industry. In Italy, the production guidelines established many decades ago indicate that pigs must be at least nine months old and have an SW of 160 ± 16 kg. Under such constraints, a restricted feeding practice is required with lean pig genotypes [10]. However, this results in low feed efficiencies, which are usually in the order of 0.28 ± 0.04 for pigs growing between 30 to 170 kg BW [24,30]

In recent times, such constraints have become progressively inadequate, and today over 15% of pigs at the age of 9 months are too lean for the needs of the ham industry [8]. Increased adiposity can be achieved in different ways—for example, with the use of pig genotypes with a high ability for fat deposition, with an increase in the dietary energy/protein ratio, the energy intake of the pigs and in the SW [5,15]. The consortia for the protection of national dry-cured hams, under the domain of the Protected Denomination of Origin (PDO), proposed a revision of the guidelines permitting carcass weights in the range 120–168 kg—but still, the pigs must be nine months old at slaughter. The result of the current research raises the question of whether younger subjects with adequate fat covering could be suitable for high-quality dry-cured ham production [5]. In any case, it is expected that the production system will evolve towards an increase in SW. 

### 4.1. Growth Performance and Feed Efficiency

In the current research, pigs were slaughtered at 230 d (7.7 months) and 258 d (8.6 months) of age, and the SW ranged from a minimum of 137 to a maximum of 225 kg. With increasing SWC, the frequency of pigs slaughtered at younger ages decreased, and that of pigs slaughtered at older ages increased. This partial confusion between age and SW was accepted, as it may become representative of the commercial conditions in the case of application of the innovative rearing strategies proposed in Malgwi et al [5]. Under current conditions, the age of slaughtered pigs is controlled by looking at the tattoo on the piglet’s skin, applied within a week from birth. The tattoo reports only the month of birth, so that piglets born towards the end of a month can be slaughtered at the beginning of the ninth month. In this way, the age at slaughter would be some days less than 270 d.

The results of the current experiment can be compared with others [10,31] achieved on heavy pigs fed restrictively in the same 90−170 kg BW interval. The pigs of these authors consumed on average 2.5−2.6 kg/d of feed, they grew on average 0.66−0.73 kg/d and the resulting gain: feed ratios were in the order of 0.253−0.284. The feed efficiency found by these authors was similar to that found in the current experiment, suggesting that there could be benefits from moving from a restricted to an ad libitum feeding practice. This would result in pigs with greater SW and greater carcass and ham adiposity, without a loss of feed efficiency compared to conventional practice.

The first relevant finding of the current paper is that feed efficiency was not related to the increase in SW. This is in apparent contradiction with the literature, which reports that feed efficiency decreases with increasing physiological maturity [1]. These authors reviewed 25 studies involving pigs harvested at weights greater than 125 kg. They found that with increasing SW and age at slaughter, there was a linear decrease in feed efficiency (gain: feed). The magnitude of this change was −0.011 per 10 kg SW increase. [1] stated that the decrease in feed efficiency can be attributed to accelerated fat accretion, declining rates of water and protein deposition, and increased maintenance requirements in heavy finishing pigs. In the current experiment, feed efficiency was not related to SW, because the heavier pigs were also those that attained greater feed intake, and a greater rate of growth. Pigs with greater SWs had greater energy and nutrient intake, so that a lower proportion of energy was partitioned towards the maintenance and a greater proportion toward the growth of the body’s constituents. This result was consistent with the results of [7], where it was found that an increased growth rate was positively related to an increase in feed efficiency (gain: feed).

The pigs in the current research evidenced good potential for growth at heavy BWs, both for lean and fatty tissues. Besides growth and residual feed efficiency, the breeding goal of this line includes traits related to the quality of raw hams [16] and their suitability for dry-curing [17]. Considering the breeding goals and the results obtained here, it may be suggested that the pigs of this line have good aptitudes for lean gain over extended ranges of BW, but also fat accretion. However, it should be considered that positive or negative relationships between feed efficiency and SW could depend on the pig genotype, due to different energy partitioning among maintenance, protein and lipid accretion throughout growth.

### 4.2. Carcass Traits

**Figure 3:**
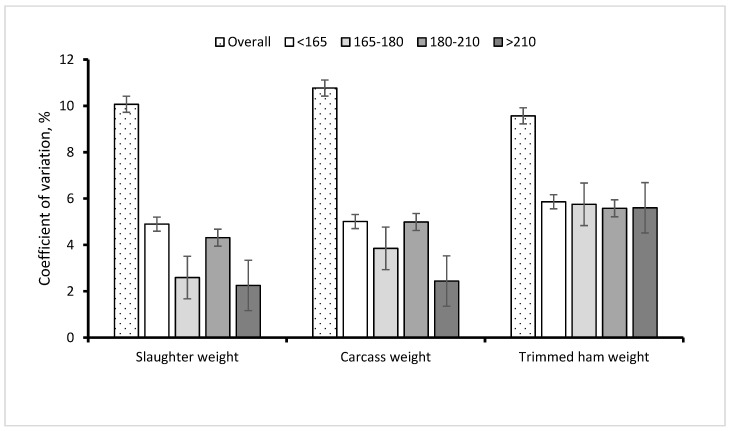
Coefficients of variation of slaughter weight, carcass weight and trimmed ham weight computed for each class of slaughter weight (<165, 165–180, 180–210, >210 kg SBW) and overall.

The proposal of new guidelines for dry-cured ham production indicates that carcass weights must range between a minimum of 120 and a maximum of 168 kg. In our research, 10 out of 159 carcasses (6.2%) were heavier than the upper limit. This would suggest that, when fed ad libitum, some Goland C21 pigs would be heavier than the maximum indicated for dry-cured ham production. As an anticipation of the age at slaughter might be not permitted by the guidelines, this shortcoming would be resolved by introducing a mild feed restriction or practices of precision feeding, resulting in benefits in terms of uniformity (Figure 3) of the pigs at slaughter.

In the current experiment, the coefficient of variation for carcass weight was 11%, whereas in previous research, where pigs were kept on restricted feeding regimes, the coefficient of variation was in the order of 6−7% [10]. Herein, carcass yield ranged from 81.8 to 83.0%. These values are comparable with those frequently found in heavy pigs [32,33]. Several studies have found increases in carcass weight more than proportional to the increase in SW, resulting in increased carcass yield [31]. In the present research, carcass yield increased in the order of 0.20% per 10 kg increase in SW—a value lower, but comparable to that found by [1], who found an increase in carcass yield of an average of 0.40% per 10 kg of SW increase, but with an impressively large standard deviation (0.31%).

The correlation between carcass weight and carcass yield was appreciable and positive (r = 0.45; *p* < 0.01). Such an increase in carcass yield is due to the differential development of the carcass fatty and lean tissues compared to the non-carcass parts [34]. In agreement with previous reports [13,34,35], the weights of the fatty cuts, namely those of back fat and belly, increased at a rate greater, while the weights of the lean tissues increased at a rate lower than that of the carcass weight. Due to the changes in the relative rate of fat and lean tissue accretion during the finishing period of the pigs, a substantial change in carcass composition occurred. The lean meat percentage, estimated from the CSB-system images, decreased linearly from 51.7 to 42.8%. The magnitude of this decrease was remarkable, as it averaged 1.47% for a 10 kg increase in carcass weight (r = 0.53; *p* < 0.01). 

The guidelines for dry-cured ham production indicate that the lean meat percentage must range between 40 and 55% [6]. In the current dataset, it was found that despite the ad libitum feeding and the heavy SW, nine pigs (5.7%) still had a lean meat percentage >55%, being too lean for the needs of the ham industry. Five of these nine pigs were slaughtered at 230 d old (161 kg SLW, on average), and only four were slaughtered at 259 d of age (169 kg SW, on average). It was concluded that an increase in SW can be considered as one of the most important ways to decrease the lean meat percentage.

### 4.3. Commercial Cuts

Information on the changes in the yields of primal cuts at different SWs is required for the analysis of pig production and the optimisation of profits. The yields of the various cuts are difficult to compare with other research, because of the different dissection procedures at slaughter, different pig genotypes and different ranges of SW, according to market demand. However, it was observed that the yields of lean cuts in the current research were slightly lower than those of pigs slaughtered at the traditional 170 kg SW. In fact, in previous research, the yields of total lean cuts, shoulder and trimmed hams, averaged 521–630, 104–140 and 215–259 g/kg, respectively [10,24,36]. 

The lower yields of lean cuts were expected because of the heavier SW and the ad libitum feeding regime of the pigs in the current research compared to the traditional restrictively fed pigs. The weights and the yields of the fatty cuts increased with increasing SW, while the weights of the loins plus ribs, shoulder, and trimmed ham increased with increasing SW, but the corresponding yields decreased. The review of [1] suggests that the loin, shoulder and ham yields decrease on average by 0.13, 0.16 and 0.17% per 10 kg of SW increase, while that of belly increases by 0.32%. The magnitude of these trends in variation is comparable to that found for the pigs in the current research, where an increase of 10 kg of SW was associated with reductions of 0.218, 0.133, 0.164 and 0.223% of the loins plus ribs, shoulders, green hams and trimmed hams yields, respectively. 

### 4.4. Ham Traits

As expected, the weight of the ham, trimmed at the slaughterhouse or the ham factory, increased with increasing SW. The weight of the trimmed ham at the slaughterhouse ranged, on average, from 12.3 to 16.4 kg, within the 12.0−18.0 kg range indicated by the proposal of the new production guidelines. However, there were seven hams (4.4%) lighter than the minimum required to achieve the label. The weight of the trimmed ham was further reduced according to the additional trimming procedure conducted at the local ham factory.

The ham weight and size, together with the inter-and intramuscular fat content, the thickness of the subcutaneous fat and the lean meat content of the hind leg, represent the main factors that can also influence the aptitude of the ham to adsorb salt [37]. It is commonly assumed that heavier hams are characterized by better seasoning properties, because of lower seasoning losses [28]. However, previous experiments have found little or no correlation between ham weight and seasoning losses [4,38]. Thus, the greater seasoning aptitude of the heavier hams was attributed to the greater adiposity of the hams harvested from older and heavier pigs [27,28]. These authors suggested that the most relevant factor affecting seasoning losses is the fat thickness, which serves as a barrier for water evaporation during seasoning.

In the current experiment, the increased SW had little influence on the majority of the ham’s quality traits, except on the subcutaneous fat depth, corresponding to the *semimembranosus* muscle, and on the roundness—a measure of muscularity. Interestingly, with increasing SW, the subcutaneous depth of the carcass increased, but the subcutaneous fat depth of the ham increased only in correspondence to the *semimembranosus* muscle, and not in correspondence to the *biceps femoris*. This seems to not be fully consistent with the results of [39], who found that the ultrasound fat thickness, measured in living pigs, was most correlated with the subcutaneous fat thickness in correspondence to the *biceps femoris* muscle (r = 0.53), rather than to the *semimembranosus* muscle (r = 0.18). In the current experiment, there was no correlation between the average carcass fat thickness and the measures of subcutaneous fat thickness taken at the two positions, with simple correlation coefficients ranging from 0.06 to 0.02. However, in agreement with previous research [17,39], the subcutaneous fat depth in correspondence to the *semimembranosus* muscle was much thinner than that measured in correspondence to the *quadriceps femoris* muscle. As the thickness of subcutaneous fat influences salt penetration and water seasoning losses during seasoning [28], measurements taken at the *semimembranosus* muscle may exert a critical role in determining the dry-curing aptitudes of ham [17]. This result suggests that with an increase in SW, the seasoning aptitude of the ham might be improved without increasing the thickness of the fat layer in correspondence to the *biceps femoris* muscle, which may not be desired by the consumer and may limit the marketability of the ham [40]. 

The influence of a thicker round shape on the seasoning aptitude of the hams, or globosity, is poorly described in the literature. In practice, a greater ham roundness is frequently associated with excessive leanness, scarce subcutaneous fat covering, greater water content and salt absorption, greater seasoning losses and poor final quality of the seasoned ham [41]. In the case of the San Daniele Consortia, shortcomings associated with the roundness assume minor relevance because the tights are pressed [42]. In the current research, an increase in SW was associated with an increase in the roundness score. Considering that the optimal roundness is between one and two over a range from one to four, the number of pigs with a round shape score of three and four was notable; 33 (21%) and 6 (3.8%), respectively. It is not possible to indicate if this increase in roundness would result in greater difficulties in controlling the seasoning process, and therefore if this will require some adjustments in the manufacturing process. On the other hand, a greater roundness would have a less negative impact if associated with greater subcutaneous fat covering at the P2 position. This issue will merit future research efforts. 

### 4.5. Sex Effects

In the Italian heavy pig production system, previous research has found little differences between gilts and barrows [43]. Such a finding could be attributed to the practice of feed restriction that could have reduced the exploitation of sexual dimorphism. In planning this research, and based on previous research [7,27,44], it was expected that the emersion of greater differences between gilts and barrows due to ad libitum feeding would permit better exploitation of inherent genetic differences. Such an expectation was confirmed, as barrows were 3.9% less efficient (gain: feed) and they had a 0.9% greater carcass yield, with a greater yield of fat and a lower yield of lean cuts. Such findings agree with those of previous researches [3,29,45]. However, in the cited literature, the differences between barrows and gilts were more accentuated, as the barrows showed 16−17% greater feed intake, 8–13% greater average daily gain, 22−27% greater backfat depth, 3−5% lower gain: feed ratio and 3−5% lower ham yield than gilts.

Some differences between barrows and gilts were also found for some subjective scores, as barrows scored lower for hemorrhages and greater for visible marbling compared to gilts. However, the magnitude of these differences was modest. Therefore, it appears that no solid reasons can be given, at this point in time, to indicate that barrows are better than gilts when intended for Italian dry-cured ham production. This is dependent on the pig genetic line, as in other productive contexts, barrows have been found to be better than gilts when intended for dry-cured ham production [24].

## 5. Conclusions

In conclusion, pigs with greater SWCs had greater average daily gain and feed consumption with similar feed efficiency, greater ham weight, muscularity and fat covering in correspondence to the *semimembranosus* muscle. Greater ham weight and fat covering in correspondence to the *semimembranosus* muscle are desired by the dry-cured ham industry for better curing aptitudes. Barrows produced hams with greater weight and marbling than gilts. A greater marbling is desired because of its positive influence on the flavor and the visual traits of green ham at the time of its selection for dry-curing. These characteristics are evaluated by the dry-cured ham industry before the curing process for better profitability and consumer acceptability of the seasoned product. Data from this research also indicate that pigs of the Goland C21 genotype can reach the traditional weight of 160 ± 16 kg at only 8 months of age—one month less than the traditional age. New knowledge about the influence of slaughter age on the seasoning aptitude of the hams, not confounding SW with slaughter age, is desired.

## Figures and Tables

**Table 1:** Ingredient composition (g/kg) of the high protein feeds used in early (90 to 120 kg BW) and late (>120 kg BW) finishing.

Ingredient	Early Finishing (90 to 120 kg Body Weight)	Late Finishing (120 kg Body Weight upwards)
Corn grain	361.8	398.9
Wheat grain	240.0	238.0
Barley grain	100.0	100.0
Soybean meal 48% (solv. ex.)	196.0	143.0
Wheat bran	26.5	7.5
Wheat middlings	-	40.0
Cane molasses	20.0	22.5
Lard	20.0	20.0
Dried sugar beet pulp	-	-
Calcium carbonate	15.0	13.0
Dicalcium phosphate	4.5	2.0
Sodium chloride	3.0	3.0
Sodium bicarbonate	2.5	2.5
Vitamin and mineral premix ^a^	2.0	2.0
Grapeseed meal	7.0	7.0
Choline, liquid, 75% ^b^	0.5	0.3
L-Lysine ^c^	1.0	0.3
DL-Methionine ^d^	0.2	0.1
L-Thryptophan, 49% ^e^	-	-

^a^ Providing per kilogram of feed: vitamin A, 8000 IU; vitamin D3, 1200 IU; vitamin E, 8 mg; Vitamin B7, 0.08 mg; vitamin B12, 0.012 mg; niacin, 16.0 mg; biotin, 8 mg; iron, 170 mg; zinc, 117 mg; copper, 14 mg; cobalt, 0.11 mg; iodine, 0.06 mg; manganese, 65 mg; magnesium, 0.14 mg; selenium 10 mg; ^b^ Choline liquid 75% (Methodo Chemicals, 42017 Novellara, RE, Italy); ^c^ L-Lysine Monoclohydrate, 98.5% pure, 78% L-Lysine (Methodo Chemicals, 42017 Novellara, RE, Italy); ^d^ DL-Methionine, 98% pure min. (Methodo Chemicals, 42017 Novellara, RE, Italy); ^e^ L-tryptophane, 50% L-Tryptophane (Methodo Chemicals, 42017 Novellara, RE, Italy).

**Table 2:** Influence of sex, slaughter weight class (SWC) and sex × SWC interactions on heavy pig growth performance and main carcass characteristics.

	Class of Slaughter Weight (SWC), kg						Sex				Sex × SWC
Items	<165	165 to 180	180 to 210	>210	SEM ^1^	*p-Linear* ^2^	Gilts	Barrows	SEM ^1^	*p*	*p*
Pigs, *n*.	26	41	82	10	-	-	72	87	-	-	-
230 d-old pigs, *n*.	23	29	24	1	-	-	32	45	-	-	-
258 d-old pigs, *n*.	3	12	58	9	-	-	40	42	-	-	-
Average age at slaughter, d	235	238	249	262	-	-	246	244	-	-	-
Live performances:											
Initial body weight	86.0	95.4	97.7	105.0	3.4	<0.001	95.3	96.7	6.3	0.48	0.85
Slaughter weight (SW), kg	153.8	172.7	193.1	214.6	3.2	<0.001	182.9	184.3	5.7	0.027	0.94
Feed intake, g/d	2880	3130	3412	3835	130	<0.001	3287	3342	241	0.45	0.64
Average daily gain, g/d	821	874	959	1074	50	<0.001	944	920	96	0.42	0.18
Gain: feed	0.283	0.279	0.280	0.279	0.009	0.72	0.286	0.275	0.018	0.043	0.21
*Post mortem* performances:											
Carcass weight, kg	125.9	142.0	159.8	178.1	2.9	<0.001	150.3	152.6	5.4	0.16	0.97
Carcass yield, %	81.8	82.2	82.7	83.0	0.60	0.043	82.1	82.8	1.1	0.039	0.31
Carcass backfat depth ^3^, mm	36.2	40.6	46.3	50.4	2.3	<0.001	42.4	44.3	4.2	0.13	0.30
Lean meat g/kg	51.7	49.6	46.6	42.8	1.7	<0.001	48.0	47.4	3.1	0.53	0.023
Wholesale cuts weight, kg:											
Total cuts ^4^	91.3	103.1	115.7	128.0	2.1	<0.001	108.9	110.2	3.8	0.25	0.73
Primal lean cuts	66.2	74.2	81.3	88.5	1.6	<0.001	77.7	77.5	3.0	0.84	0.62
Primal fat cuts	25.1	28.9	34.4	39.4	1.3	<0.001	31.2	32.7	2.5	0.043	0.89
Wholesale cuts yield, g/kg:											
Total cuts ^4^	725	727	724	718	5.1	0.15	725	722	9.5	0.40	0.48
Lean cuts	526	523	509	497	7.6	<0.001	519	509	14.3	0.026	0.58
Fat cuts	199	204	215	221	7.3	0.001	206	213	13.7	0.08	0.64

^1^ Standard error; Data were from 159 pigs: 72 gilts and 87 Barrows fed ad libitum, from 133.8 to 225.1 kg BW (*n* = 159); ^2^ As the quadratic and cubic components were never significant, the corresponding *p*-values were omitted; ^3^ Average of two measures taken from the hot carcass at the points of minimum (lombar) and maximum (shoulder) backfat depth; ^4^ This measure corresponds to the sum of the weights of shoulders, hams, loins and ribs, belly and lard. Other minor cuts were not measured.

**Table 3:** Influence of sex, slaughter weight and sex × SWC interactions on heavy pig commercial cut weights and yields.

	Class of Slaughter Weight (SWC), kg						Sex				Sex × SWC
Items	<165	165 to 180	180 to 210	>210	SEM ^1^	*p-Linear* ^2^	Gilts	Barrows	SEM ^1^	*p*	*p*
Wholesale cuts, kg:											
Loins and ribs	19.1	21.4	22.9	24.8	5.9	<0.001	22.4	21.8	1.1	0.06	0.98
Shoulders	16.7	18.6	20.5	22.3	0.5	<0.001	19.4	19.6	1.0	0.44	0.42
Green hams	30.5	34.1	37.9	41.4	0.8	<0.001	35.9	36.1	1.5	0.64	0.48
Trimmed hams ^3^	24.8	27.8	30.4	32.9	0.7	<0.001	29.0	29.0	1.2	0.89	0.43
Trimming loss ^3^	5.7	6.3	7.5	8.5	0.3	<0.001	6.9	7.1	0.6	0.37	0.93
Backfat	15.7	18.0	21.0	24.4	0.7	<0.001	19.7	19.9	1.3	0.66	0.94
Belly	9.4	10.9	13.4	15.0	0.8	<0.001	11.5	12.9	1.5	0.005	0.50
Wholesale Cut Yields, g/kg Carcass:											
Loins and ribs	152	151	143	140	3.7	<0.001	150	143	6.9	0.003	0.99
Shoulders	133	131	128	125	2.7	0.010	130	129	5.6	0.72	0.59
Green hams	242	240	237	232	3.5	0.003	239	237	6.5	0.16	0.26
Trimmed legs	197	196	190	184	3.3	<0.001	194	190	6.1	0.11	0.33
Ham trimming loss ^3^	44.9	44.3	47.1	47.5	1.9	0.09	45.9	46.1	3.6	0.83	0.91
Backfat	124	127	131	137	3.8	<0.001	131	129	6.7	0.61	0.88
Belly	75	77	84	84	5.3	0.04	75.6	84.0	9.9	0.006	0.22

^1^ Standard error; Data were from 159 pigs: 72 gilts and 87 Barrows fed ad libitum, from 133.8 to 225.1 kg BW; ^2^ As the quadratic and cubic components were never significant, the corresponding *p*-values were omitted; ^3^ Data are from ham trimming at the slaughterhouse (SH).

**Table 4:** Influence of sex and slaughter weight on the characteristics of trimmed legs for dry-cured ham production.

	Class of Slaughter Weight (SWC), kg						Sex				Sex × SWC
Items	<165	165 to 180	180 to 210	>210	SEM ^1^	*p-Linear* ^2^	Gilts	Barrows	SEM ^1^	*p*	*p*
Trimmed ham, kg											
slaughter house (SH)	12.3	13.8	15.1	16.4	0.3	<0.001	14.4	14.4	0.6	0.96	0.44
ham factory (HF) ^3^	11.9	13.2	14.4	15.7	0.3	<0.001	13.8	13.8	0.6	0.86	0.65
losses at the HF ^3^, kg	0.44	0.58	0.68	0.70	0.07	<0.001	0.58	0.62	0.14	0.33	0.21
losses at the HF ^3^, g/kg	35.6	42.0	44.5	43.2	0.49	0.11	40.1	42.5	0.93	0.41	0.39
Green ham quality traits:											
Round shape ^4^	1.35	1.58	1.95	2.57	0.40	0.002	2.04	1.68	0.76	0.12	0.20
Veining ^5^	1.73	1.64	1.43	1.12	0.41	0.12	1.40	1.57	0.76	0.45	0.95
Haemorrhage ^6^	0.15	0.37	0.25	0.50	0.22	0.23	0.46	0.17	0.46	0.037	0.12
Visible marbling ^7^	0.84	0.90	0.73	0.73	0.33	0.65	0.56	1.04	0.61	0.011	0.14
Meat colour ^8^	−0.63	−0.69	−0.64	−0.56	0.61	0.88	−0.81	−0.44	1.14	0.29	0.28
Fat cover score ^9^	0.20	0.41	1.09	1.20	0.69	0.09	0.71	0.74	1.28	0.94	0.52
Subcutaneous fat, mm											
P1 position ^10^	29.5	30.6	34.1	32.6	3.1	0.20	30.6	32.7	5.9	0.23	0.92
P2 position ^11^	6.6	6.4	7.0	7.7	0.4	0.005	7.05	6.83	0.8	0.39	0.35

^1^ Standard error; Data were from 159 pigs: 72 gilts and 87 Barrows fed ad libitum, from 133.8 to 225.1 kg BW; ^2^ As the quadratic and cubic components were never significant, the corresponding *p*-values were omitted; ^3^ At arrival at the ham factory (HF), the hams were trimmed again and weighted; ^4^ Round shape (0 = very flat to 4 = very round; 1–2 optimum); ^5^ Veining (0 = absent to 4 = evident, 0 = optimum); ^6^ Haemorrhage (0 = absent 3 = evident, 0 = optimum); ^7^ Visible marbling (0 = absent to 4 = every evident, 1–2 = optimum); ^8^ Meat colour (−4 = pale to 4 = dark, 0 = optimum); ^9^ Fat cover score (−4 = thin to 4 = thick); ^10^ Ham subcutaneous fat depth measured at the point of minimum depth in the proximity of m. *biceps femoris* with a ruler; ^11^ Ham subcutaneous fat depth measured in proximity of m. *semimembranosus* with a portable ultrasound system (Aloka SSD 500 equipped with UST-5512 7.5 MHz linear transducer probe, Hitachi Medical Systems S.p.A., Milan, Italy).

## Data Availability

The data supporting the findings of this study are available from Gorzagri s.s., but restrictions apply to the availability of these data, which were used under license for the current study and are not publicly available. Data are however available from the authors upon reasonable request and with the permission of Gorzagri s.s.
